# Treating subclinical hypothyroidism in individuals with or without mental health problems –A Delphi based expert consensus study in two countries

**DOI:** 10.3389/fendo.2023.1204842

**Published:** 2023-07-12

**Authors:** Ingrid Lieber, Christina Maria Van Der Feltz-Cornelis, Salman Razvi, Andrew S. Moriarty, Scott Wilkes, Michael Ott, Julie Mannchen, Mats Eliasson, Ursula Werneke

**Affiliations:** ^1^ Department of Clinical Sciences, Division of Psychiatry, Sunderby Research Unit, Umeå University, Umeå, Sweden; ^2^ Department of Health Sciences, University of York, York, United Kingdom; ^3^ Hull York Medical School, University of York, York, United Kingdom; ^4^ York Biomedical Research Institute, University of York, York, United Kingdom; ^5^ R&D Department, Tees Esk and Wear Valleys NHS Foundation Trust, Darlington, United Kingdom; ^6^ Institute of Health Informatics, University College London, London, United Kingdom; ^7^ Translational and Clinical Research Institute, Newcastle University, Newcastle upon Tyne, United Kingdom; ^8^ Faculty of Health Sciences and Wellbeing, School of Medicine, University of Sunderland, Sunderland, United Kingdom; ^9^ Department of Public Health and Clinical Medicine – Medicine, Umeå University, Umeå, Sweden; ^10^ Department of Public Health and Clinical Medicine, Family Medicine, Umeå University, Umeå, Sweden; ^11^ Department of Public Health and Clinical Medicine, Sunderby Research Unit, Umeå University, Luleå, Sweden

**Keywords:** subclinical hypothyroidism, TSH, affective disorder, Delphi method, consensus, practice guideline, thyroxine, diagnosis

## Abstract

**Background:**

Subclinical hypothyroidism (SCH) is a common endocrine problem with prevalence estimates between 4% and 20%. Symptoms are often non-specific but can substantially affect well-being leading to repeated medical consultations. The effect of thyroid hormone replacement therapy (THRT) in patients with SCH remains uncertain. Current guidelines, limited by the lack of high-quality evidence, have been controversial with limited adherence in clinical practice.

**Methods:**

Three-round modified Delphi method to establish consensus regarding diagnosis and treatment of individuals with SCH with and without affective disorder or anxiety, conducted with clinicians from three specialties, general practice, endocrinology and psychiatry, and two countries, Sweden and the United Kingdom.

**Results:**

Sixty clinicians, 20 per specialty, were recruited. Fifty-three (88%) participants completed all three rounds. The participants reached consensus on five of the 26 practice statements that (a) repeated testing was required for the diagnosis of subclinical hypothyroidism, (b) antibody screening should usually occur, and (c and d) antibody screening would strengthen the indication for thyroid hormone replacement therapy in both individuals with or without affective disorder or anxiety. The participants disagreed with (e) a requirement of a TSH threshold ≥ 20 mIU/L for thyroid hormone replacement therapy start. Psychiatrists and GPs but not endocrinologists, agreed that there was a frequent discrepancy between laboratory results and clinical symptoms, and disagreed that testing for thyroid dysfunction was overused in patients presenting with depression or anxiety, or fatigue.

**Conclusions:**

In many aspects, attitudes toward diagnosing and treating SCH remain diverse. The inability of our Delphi panel to achieve consensus on most items and the disagreement with a TSH ≥ 20 mIU/L threshold for treatment suggest that the concept of SCH may need rethinking with a better understanding of the hypothalamic-pituitary-thyroid physiology. Given that the scientific evidence is currently not conclusive, guidelines in this area should not be taken as definitive.

## Introduction

Subclinical hypothyroidism (SCH) is a common endocrine problem, characterised by elevated concentrations of serum thyroid stimulating hormone (TSH) and serum free thyroxine (fT4) concentrations within its reference range. Depending upon the population sampled, prevalence rates between 4% and 20% have been reported. Age, sex, body mass index, ethnicity, iodine intake, thyroid peroxidase antibody (TPOAb) status, and TSH cut-off point are among the factors that can affect prevalence estimates (1). Symptoms attributed to SCH are often non-specific, including tiredness/fatigue, cold intolerance, weight gain, cognitive dysfunction, depression, and anxiety (1–3). These symptoms can significantly affect well-being, leading to repeated medical consultations, request for inappropriate investigations, and dissatisfaction with treatment (3).

The effect of thyroid hormone replacement therapy (THRT) in patients with SCH remains uncertain. One meta-analysis of THRT in individuals with SCH published in 2018 did not find any improvement in general quality of life or thyroid related symptoms (4) This meta-analysis included two randomised controlled trials (RCT) with individuals of at least 65 years or older, accounting for 38% of the pooled sample (5, 6). Others pointed then out that based on these findings, treatment might be erroneously denied to younger or symptomatic patients (7). In 2019, a clinical practice guideline strongly advised against treatment of SCH, unless the TSH concentration exceeded 20 mIU/L. The guideline did not apply to women who were pregnant or women trying to become pregnant. The guideline might also not apply to young adults, i.e., less than 30 years old, or patients with severe symptoms (8). However, this practice guideline raised concerns from doctors and patients alike (9). For instance, a treatment threshold of a TSH of 20 mIU/L might deny some individuals a treatment they could benefit from, particularly younger patients. The guideline might also place an undue weight on biochemical abnormalities, rather than considering an individual in his/her entirety. Finally, a psychiatric perspective was lacking. Furthermore, characterizing the decision to use THRT as a binary and binding choice would inappropriately simplify the way clinicians interacted with these patients (7). These discussions show that treatment of SCH remains controversial with substantial cause for contention between doctors and patients. In addition, TSH elevations may be spontaneously reversible in a large proportion of individuals with SCH. One prior cohort study had shown that within five years, TSH concentrations had normalised in 62% individuals with initial concentrations between 5.6 and 10 mIU/L, and in 27% individuals with an initial TSH concentration of > 10 mIU/L (10). Subjective symptoms and illness perception may not match “objective” clinical findings and biochemical abnormalities. An overreliance on TSH as a sole marker of wellbeing may result in clinical symptoms being ignored. Conversely, overreliance on symptoms may lead to inappropriate THRT prescribing and may lead to over-treatment at the patient’s request (11). In individuals with mood disorders, it remains unclear how SCH and treatment or lack of treatment thereof affects mental status. Guidelines can only ever complement clinical intuition and patients’ wishes, particularly when there is limited high-quality evidence to guide clinical decision-making. In view of the continued controversy and the inadequate available evidence, a Delphi panel study with experts from three different specialties in two countries was undertaken.

The overall aim of this study was to explore attitudes toward clinical practice regarding SCH treatment in individuals with or without affective disorder or anxiety. Specifically, we aimed at exploring the attitudes towards THRT for SCH from representatives comprising three medical “stakeholder” specialties: general practice, endocrinology, and psychiatry, from two countries with similar health care systems, the United Kingdom (UK) and Sweden. We also aimed to determine whether a consensus could be reached regarding THRT use for SCH with or without affective disorder or anxiety.

## Method

### Study design

The study used a modified Delphi method to establish consensus regarding diagnosis and treatment of individuals with SCH with or without affective disorder or anxiety. Our modified Delphi study consisted of two stages and three consensus-building rounds. The study was a collaboration between three universities in the UK: the University of York (CMvdFC, AM), Newcastle University (SR), and University of Sunderland (SW); and Umeå University, in Sweden (IL, MO, JM, ME, UW). The study was carried out in the UK and Sweden between February and September 2022.

### Ethics and consent

The study protocol was assessed by the Swedish Ethical Review Authority and the Department of Health Sciences research governance and ethics review board of the University of York, both of which waived the need for ethical approval given the Delphi design of the study, since panellists participated only in a professional capacity. Panellists consented verbally or *via* e-mail at the recruitment stage, and then re-confirmed their consent electronically at the beginning of round one.

### Sample

The experts for the Delphi panel were recruited by the core research group according to pre-determined criteria, (a) accredited specialists from the three relevant specialties, psychiatry, endocrinology, or general practice, (b) engagement with thyroid problems in clinical practice, (c) practice or comprehensive understanding of practice in the participating countries, and (d) capacity and willingness to participate and dedicate time to the study. To maximise the value for real-life clinical practice, we deemed engagement in clinical work more important than engagement in academic activities. To maximise the response rate, we used a convenience sample based on the research group’s knowledge of experts regionally and/or nationally. To minimise the risk of selection and dominance bias, we recruited experts from two different countries with a similar health care system but no previous inter-country communication. To further minimise bias, members of the core research group were not eligible for participation in the Delphi panel and the experts participated in the surveys anonymously. We aimed to recruit 60 panellists to the expert panel: 30 from each country and 20 from each specialty. Based on an assumed drop-out rate of 20% our sample size would be sufficiently large to achieve the minimum recommended number of experts for a Delphi panel of 10 participants (12) for each specialty from both participating countries with a good margin.

### Survey procedures

**Figure 1:**
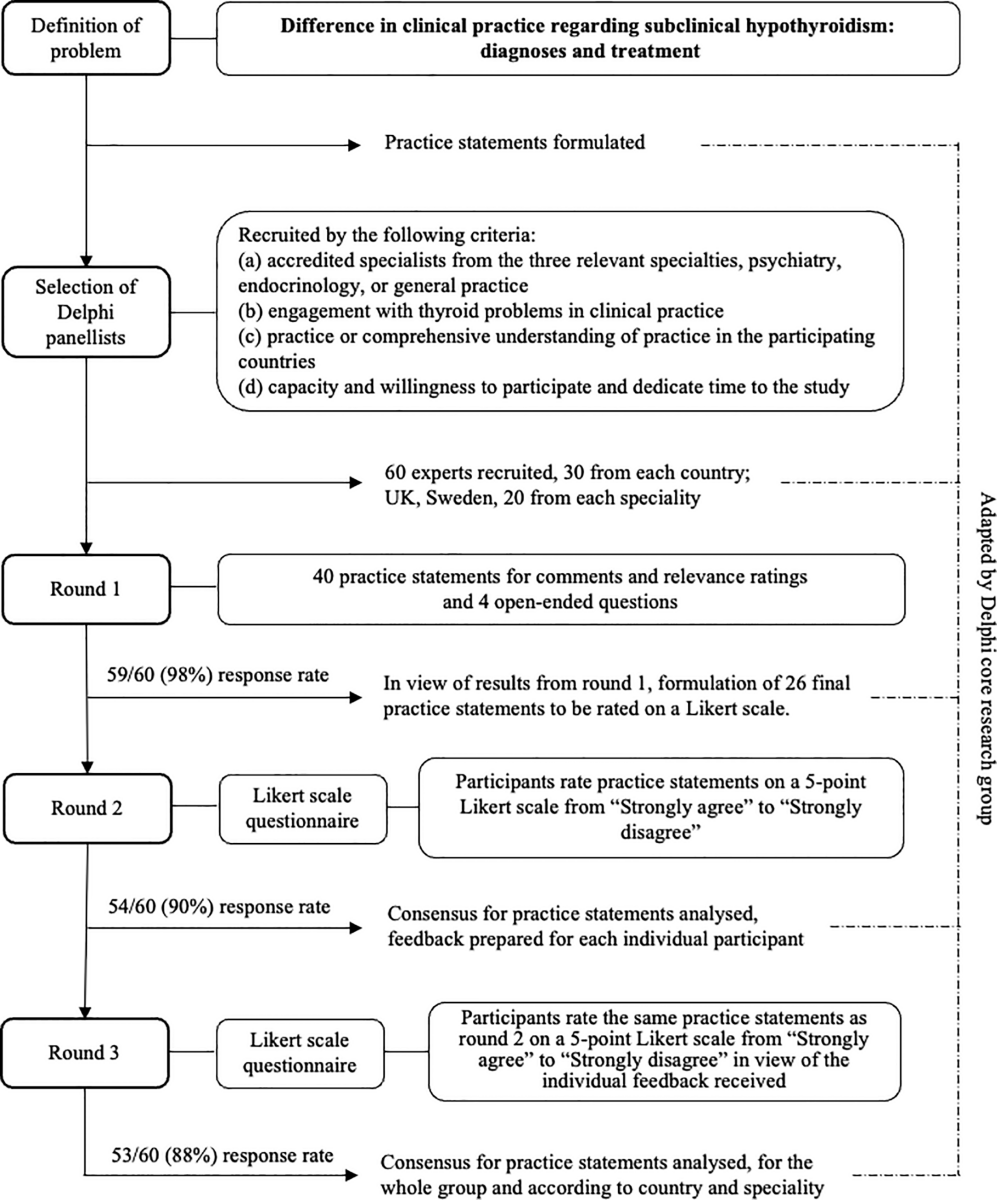
The Delphi consensus process.

We conducted the study in two stages (i), creation of the practice statements (PS) to be submitted to the Delphi panel, and (ii) the actual Delphi consensus building process (Delphi process) (**Figure 1**). In the first stage, we as the author group identified the topics to be examined and created the first set of PS to be used in the first round of the Delphi process. Forty PS were created concerning diagnosis and treatment of SCH. At this stage, it was also defined to which group of individuals the PS would not apply, children, adolescents, pregnant women, or women trying to become pregnant. The PS were then transferred to an electronic questionnaire using the online survey software Webropol (**Appendix**). In the second stage, the Delphi panel assessed and rated the PS in three rounds.

### Round 1

The PS were then submitted to the panellists. First, the panellists were asked to rate the relevance of each PS on a visual analogue scale from 0% (not clinically relevant at all) to 100% (very clinically relevant). Then, the panellists were asked to comment on each PS regarding whether it should be changed and if so, how. At the end of round 1, the principal researcher created a summary report for the revision of the PS. The PS were removed when they had received a mean relevance score of less than 30%. This cut-off point was chosen to reflect that the three involved specialties may have different priorities. Then, the author group revised the PS in light of the feedback received. The revised questionnaire contained 23 PS. For the PS, 11, 15, 17, parallel forms were created to apply to individuals with or without affective disorder or anxiety. Therefore, the final number of PS was 26 (**Appendix**).

### Round 2

The revised PS were then resubmitted to the Delphi panel. The panellists were now asked to rate the PS on a five-point Likert scale from “strongly agree” to “strongly disagree”. After conclusion of this round, the principal researcher analysed the distribution of scores and prepared individual feedback to each panellist. The feedback provided a graphic distribution of responses for the whole group of panellists in comparison to the panellists` own response for each PS.

### Round 3

The panellists received their feedback and were then asked to rate the same PS as round 2 in view of the feedback.

### Achievable strength of guidelines

In a final step, we explored the achievable strength of guidelines in light of the findings of our consensus panel, using four determinants suggested by the Grading of Recommendations Assessment, Development, and Evaluation (GRADE) working group (13).

### Statistical analysis and consensus

We analysed the responses from rounds 2 and 3 descriptively, establishing the proportion of agreement for each PS (median, IQR). Statements were ranked according to percent agreement reached and tendency to change based on feedback. Consensus is defined in positive and negative terms as 75% agreement (strongly agree or agree) or 75% disagreement (strongly disagree or disagree). We used a 75% cut-off point for two reasons; (a) 75% was the median threshold to define consensus in a systematic review of 98 Delphi studies (14), and (b) 75% agreement has been shown necessary to shift dissenting opinions (15). We then analysed the results stratified by specialty or country.

### Software

For the Delphi consensus building process, we used the online survey tool Webpropol. The statistical analysis was conducted with SPSS v 27 (Chi, Ill).

## Results

We recruited 20 psychiatrists, 20 GPs, and 20 endocrinologists. Of the 60 experts, who agreed to participate in this Delphi study, 53 (88.3%) completed all three rounds. The distribution between specialties and countries remained even (**Table 1**).

**Table 1:** Participation according to specialty or country.

	Round 1	Round 2	Round 3
Total, n (%)^a^	59 (98.3)	54 (90.0)	53 (88.3)
Specialty, n (%) GP Endocrinology Psychiatry	20 (33.9) 20 (33.9) 19 (32.2)	18 (33.3) 18 (33.3) 18 (33.3)	18 (34.0) 17 (32.0) 18 (34.0)
Country n (%)^b^ UK Sweden	29 (49.2) 30 (50.8)	26 (48.1) 28 (51.9)	26 (49.1) 27 (50.9)

a Of 60 experts who had originally agreed to participate.

b Practice or comprehensive understanding of practice in the participating countries.

### Consensus reached by the whole panel

**Figure 2:**
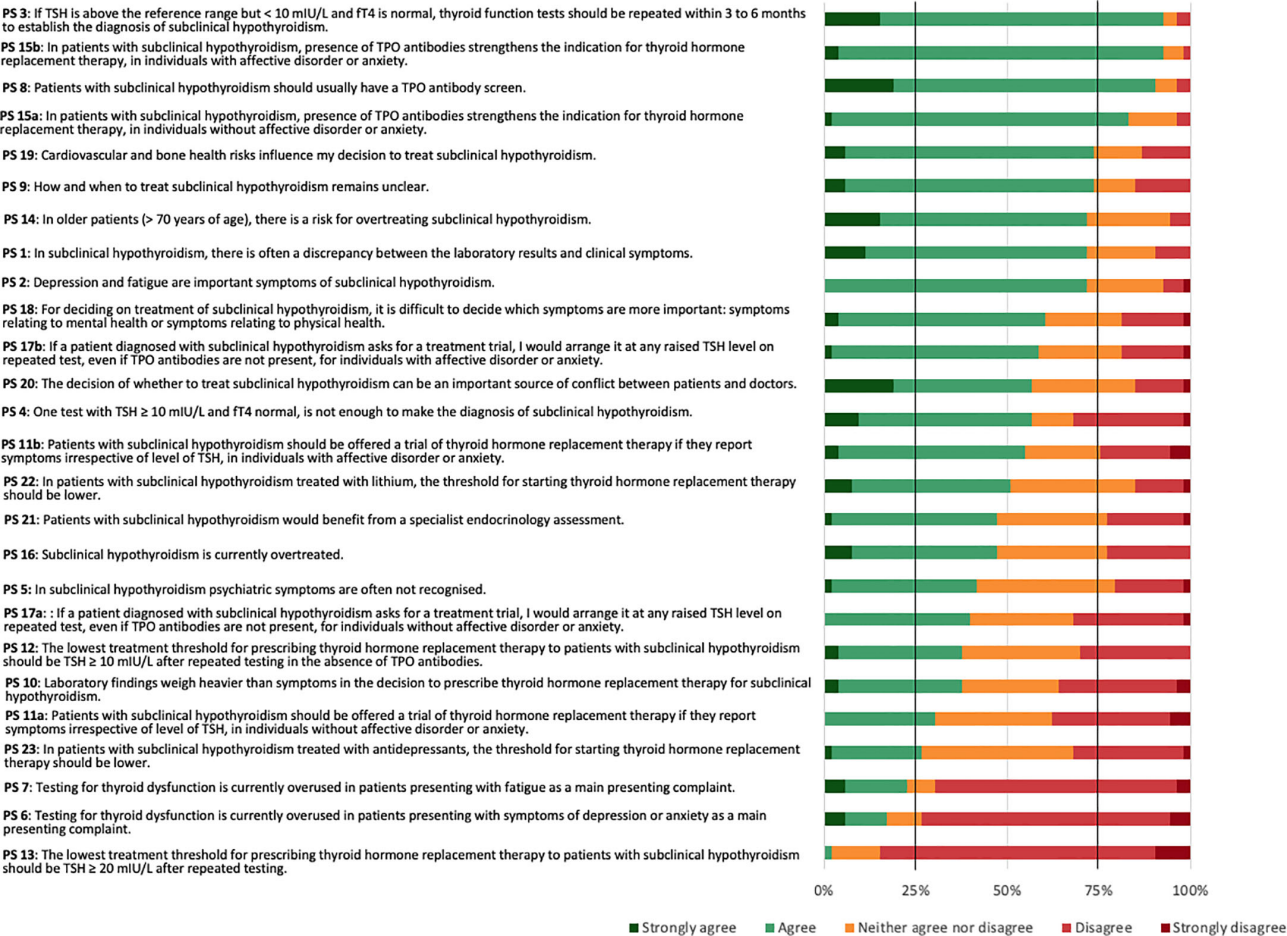
Consensus for the 26 practice statements (PS) in the whole sample.

The 53 panellists reached consensus on five (19.2%) of the 26 PS. The panellists reached a positive consensus on four (15.4%) statements and a negative consensus on one (3.8%) statement (**Figure 2**).

The PS reaching positive consensus concerned repeated testing as a means to establish the diagnosis of SCH (PS 3), need for TPOAb screen (PS 8), and TPOAb presence as an indication for THRT in both individuals without (PS 15a) or with (PS 15b) affective disorders or anxiety. Four statements narrowly missed the 75% positive consensus threshold. These concerned cardiovascular and bone health as influencing factors for the decision to treat SCH (PS 19), uncertainty about how and when to treat SCH (PS 9), a risk of overtreating SCH in older patients (PS 14), and a frequent discrepancy between laboratory results and clinical symptoms (PS 1). The statement reaching negative consensus concerned a lowest threshold of TSH ≥ 20 mIU/L after repeated testing for prescribing THRT (PS 13). One further statement narrowly missed the 75% negative consensus threshold. This concerned an overuse of testing for thyroid dysfunction in patients mainly presenting with symptoms of depression or anxiety (PS 6). The most diverse attitudes for the Delphi panel concerned PS 10: whether laboratory findings would weigh heavier than symptoms in the decision to prescribe THRT for SCH. Here, 38% agreed or strongly agreed, 36% strongly disagreed or disagreed, and 26% neither agreed nor disagreed.

### Consensus according to specialty

**Figure 3:**
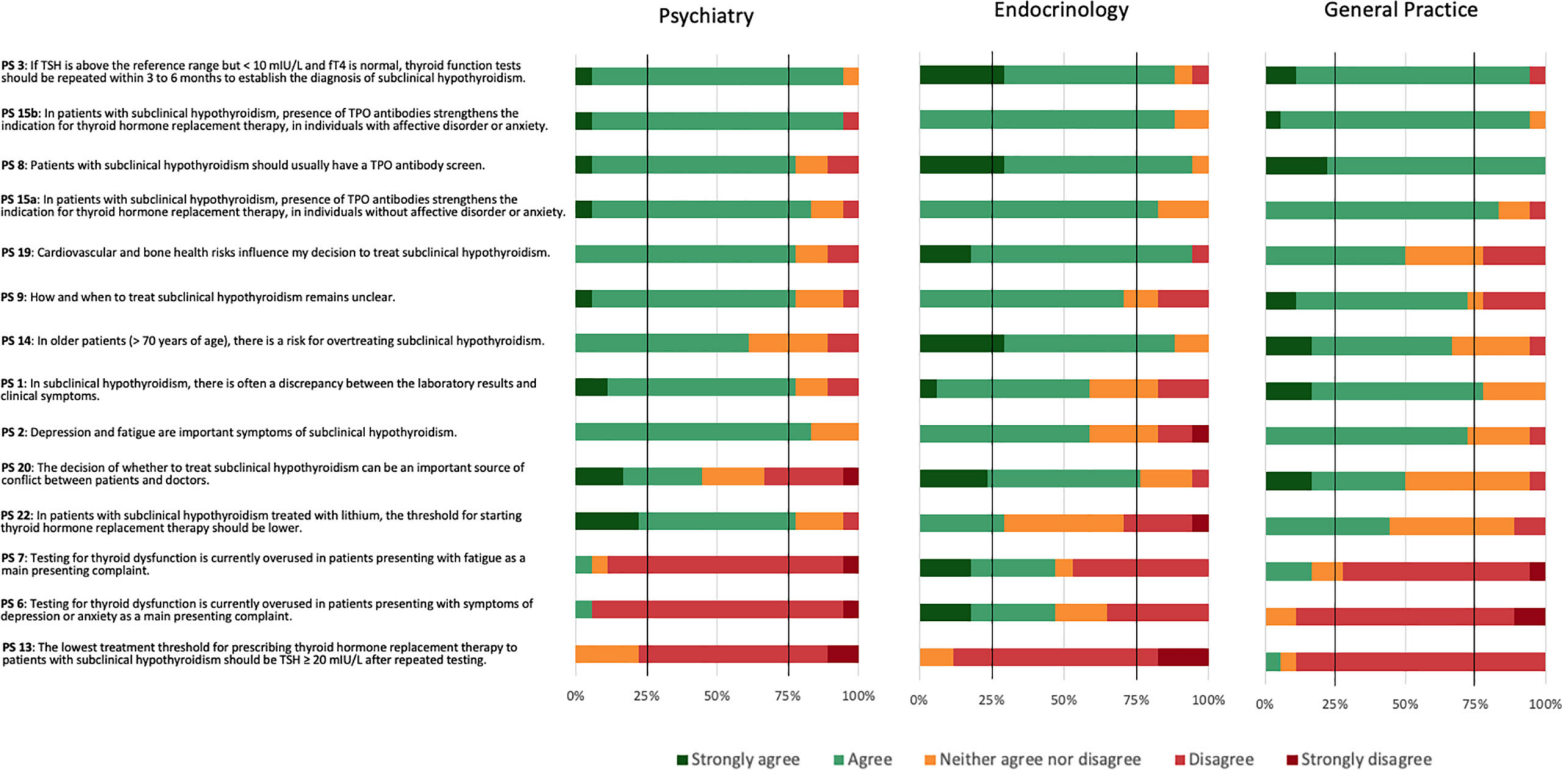
Consensus for the 26 practice statements (PS) according to specialty.

Psychiatrists reached positive consensus on nine PS (PS 1, 2, 3, 8, 9, 15a, 15b, 19, and 22), endocrinologists on seven PS (PS 3, 8, 14, 15a, 15b, 19, and 20), and GPs on five PS (1, 3, 8, 15a, and 15b) (**Figure 3**). GPs narrowly missed positive consent for two statements (PS 2 and 9). Psychiatrists and GPs, but not endocrinologists, reached positive consensus for PS 1 regarding a frequent discrepancy between laboratory results and clinical symptoms. Psychiatrists and endocrinologists, but not GPs, reached positive consensus for PS 19 that cardiovascular and bone health influenced the decision to treat SCH (PS 19). Endocrinologists and GPs felt more strongly about the need for a TPOAb screen (PS 8) than psychiatrists, although psychiatrists also agreed. Endocrinology was the only specialty achieving consensus regarding PS 20 that the decision of whether to treat or not to treat could be an important source of conflict between patients and doctors and PS 14 concerning the risk for overtreating older patients with SCH. Psychiatrists reached negative consensus on three PS (6, 7, and 13), GPs on two PS (6, 13), and endocrinologists on one PS (PS 13). All three specialties reached negative consensus regarding a lowest threshold of TSH ≥ 20 mIU/L after repeated testing for prescribing THRT (PS 13). Additionally, psychiatrists and GPs disagreed with testing for thyroid dysfunction being overused in patients presenting with depression or anxiety (PS 6) or fatigue (PS 7).

### Consensus according to country

**Figure 4:**
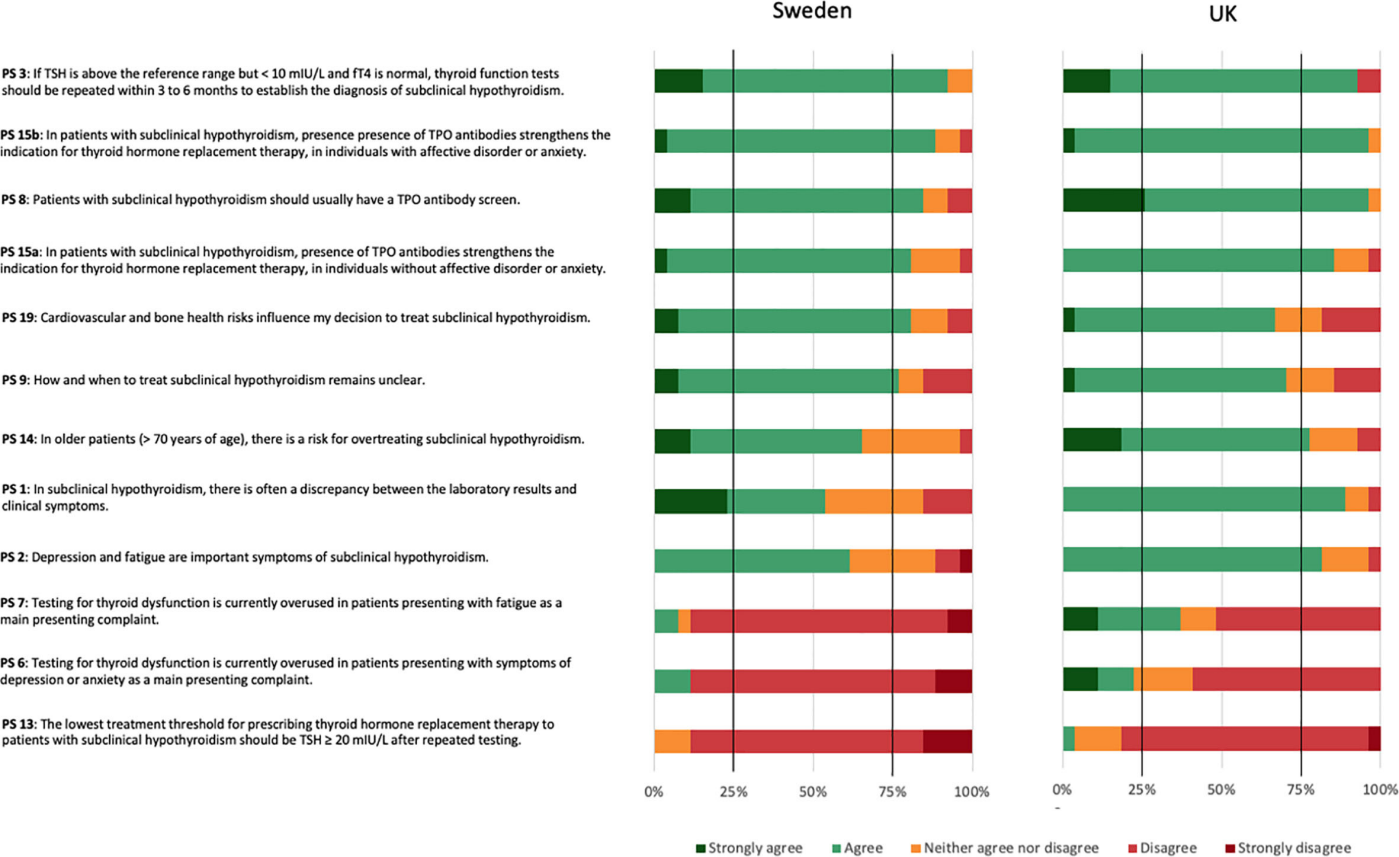
Consensus for the 26 practice statements (PS) according to country.

UK reached positive consensus on seven (PS 1, 2, 3, 8, 14, 15a, and 15b), and Sweden on six (PS 3, 8, 9, 15a, 15b, and 19) (**Figure 4**). UK, but not Swedish panellists, reached a positive consensus regarding SCH often presenting with a discrepancy between laboratory results and clinical symptoms (PS 1), depression and fatigue being important symptoms of SCH (PS 2), and the risk for overtreating older patients with SCH (PS 14). Swedish, but not UK panellists, reached a positive consensus regarding cardiovascular and bone health as influencing factors for the decision to treat SCH (PS 19). They also reached a positive consensus regarding the uncertainty about how and when to treat SCH (PS 9). UK panellists reached a negative consensus on one PS (PS 13). Swedish panellists reached negative consensus on three PS (PS 6, 7, and 13). Both country panels countries reached negative consensus regarding a lowest threshold of TSH ≥ 20 mIU/L after repeated testing for prescribing thyroid hormone replacement therapy (PS 13). Additionally, Swedish panellists disagreed with testing for thyroid dysfunction being overused in patients presenting with depression or anxiety (PS 6) or fatigue (PS 7).

### Achievable strength of guidelines

Applying the GRADE determinants to diagnosis and treatment of subclinical hypothyroidism and placing them into the context the findings of our results, suggested that recommendations in this area could only be weak at present (**Table 2**).

**Table 2:** Determinants for the achievable strength of recommendations for the diagnosis and treatment of subclinical hypothyroidism according to GRADE guidelines (based on Andrews et al., 2013) (3, 4, 13, 16–23) .

Determinant		Rating of achievable strength of recommendations for diagnosis and treatment of subclinical hypothyroidism	Determinant in the context of the findings of the Delphi study
i.	Balance between desirable and undesirable consequences of alternative management strategies *The closer the balance the less likely a strong recommendation*.	WEAK Subclinical hypothyroidism does not tend to involve life-death decisions. This increases flexibility for decision making and consideration of alternative strategies. However, adverse effects of a decision may not be apparent at the decision point but only emerge later. Decisions may not be reversed at a later point, many patients continue thyroid hormone replacement therapy for life, once started (16). Both suppressed and high TSH may adversely affect cardiovascular and bone health (17).	Positive consensus was only achieved on 4/26 items. Negative consensus was only achieved on 1/26 items regarding a treatment threshold of TSH ≥ 20 mIU/L.
ii.	Confidence in estimates of effect. *The lower the confidence, the less likely strong a recommendation*.	MODERATE Several meta-analyses exist, yielding high confidence in the estimates of effects at face value. However, a bias towards patients with milder symptoms and older patients in intervention trials compromises generalisability to patients with more severe symptoms and younger patients (4, 18–20).	Despite the available evidence and its translation into guidelines, 74% of panellists felt that how and when to treat subclinical hypothyroidism remained unclear (PS 9).
iii.	Uncertainty or variability in values and preferences. *The less the confidence in typical values and preferences, and the greater the variability, the less likely strong a recommendation.*	WEAK Subjective symptoms may differ from objective (laboratory findings), which weakens confidence in typical values (3). The individual fT4 reference range to which TSH reacts may differ from the population-based reference range. This may be genetically determined (21, 22). Rise in TSH concentrations may be a normal consequence of aging (22). Changes in TSH and fT4 may be transient and revert to normal (10, 23). The balance of risks and benefits may change with age. All factors can lead to substantial variability in preferences in patients or doctors, or between patients and doctors.	Psychiatrists achieved more consensus than GPs and Endocrinologists achieved more consensus than GPs. Psychiatrists and GPs may be more psychologically minded and more accepting of unexplained clinical symptoms reaching consensus that there was often a discrepancy between laboratory results and clinical symptoms (PS 1). This may translate in a greater willingness to prioritise subjective symptoms over objective findings (PS 1), which reduces the potential for conflict between doctors and patients (PS 20)
iv.	Resource use. *The higher the resource use, the less likely strong a recommendation.*	WEAK Thyroid function testing and thyroid hormone replacement therapy is relatively cheap compared to other health care interventions. Costs may only accumulate over time and not apparent the decision point.	Swedish panellists did not feel that testing for thyroid dysfunction was overused in patients presenting with symptoms of depression or anxiety (PS 6), or fatigue (PS 7). UK panellists did not reach a consensus for these PS. This may possibly reflect a difference in healthcare resources.

PS, Practice statement.

**Table 2**. Determinants for the achievable strength of recommendations for the diagnosis and treatment of subclinical hypothyroidism according to GRADE guidelines (based on Andrews et al., 2013) (3, 4, 13, 16–23)

## Discussion

Our findings show that the diagnosis and treatment of SCH remain an area of clinical practice in which consensus is difficult to achieve. Consensus was best regarding the need for repeated testing, usefulness of TPOAb testing for diagnosis and treatment decisions, and the unacceptability of TSH ≥ 20 mIU/L (after repeated testing) as a treatment threshold. For all respective statements (PS 3, 8, 13, 15a, and 15b), consensus was reached not only by the whole panel but also across the three specialties and the two countries involved. Psychiatrists and GPs seemed more inclined than endocrinologists to take psychological symptoms into account regarding a potential discrepancy between laboratory results. This may have led to psychiatrists and GPs perceiving a greater need for testing. Conversely, endocrinologists perceived a greater potential for conflict regarding treatment decisions. Swedish panellists felt a greater uncertainty about how and when to treat SCH than UK panellists. This may explain why Swedish panellists seemed more strongly guided by cardiovascular and bone health as influencing factors, which might be seen as “tangible” factors. This may also explain why Swedish panellists felt to a lesser extent than UK panellists that testing was overused in patients with depression or anxiety, or fatigue. Our finding that there is little consensus regarding management of SCH amongst practitioners is in line with three previous surveys. In these surveys, opinions differed on whether symptoms or presence of antibodies were most important for the decision to treat SCH. Reduction of risk factors for cardiovascular disease seemed less important (24–26).

Our findings call into question the applicability and utility of the clinical practice guideline for the treatment of SCH published in 2019 (8). This guideline, at the time, resulted in controversy, which is confirmed by the results of our Delphi study, particularly that the requirement of a treatment threshold of TSH ≥ 20 mIU/L may be unacceptable. Our findings also do not concord with the expectation of the initiators of the 2019 guidelines of “little variability in how patients weigh the lack of benefit against possible harm” (8). Our Delphi panel acknowledged a considerable amount of uncertainty regarding diagnosis and treatment of SCH, an uncertainty likely to be shared by many patients.

Creating clinical guidelines is complex. Stages in guideline development involve identifying and formulating the question to be addressed, assembling an appropriately qualified and diverse review team, collating the evidence, assessing the quality of the evidence, and finally proceeding from evidence to recommendation (13, 27). Collating and assessing the evidence is a crucial step in the formulation of guidelines. The guidelines formulated by Bekkering et al., 2019 (8), which were the starting point for our Delphi study, were mainly based on a meta-analysis failing to show any improvement in quality of life with THRT (4). Other recent meta-analyses concord that in terms of depression or quality of life, little seems to be gained from THRT in individuals with SCH (18–20). Yet, the conclusions of these meta-analyses may depend on the samples selected. Results found in older patients may not be generalisable to younger patients. This may partly be due to older patients being misclassified as SCH on the basis of higher TSH concentrations, which may be physiological in older patients (4, 18, 19). Our results for the whole panel confirmed a concern about potential overtreatment of older individuals (PS 14). This result, however, was mainly driven by the consensus achieved by endocrinologists; psychiatrists and GPs as separate groups did not achieve consensus. A concern about potential overtreatment of older individuals is supported by the TRUST trial, the largest of its kind with 737 participants with mild SCH who were at least 65 years of age. This trial did not show any consistent beneficial effect of levothyroxine treatment on thyroid-related symptoms. Changes in quality of life were marginal and fluctuated depending on the timepoint of measurement (5).

Also, clinical trials may be biased toward individuals with only mild symptoms of SCH; individuals with more severe symptoms may already have been offered THRT (4). This could explain the discrepancy between the published scientific evidence, biased towards mild presentations, and the perceptions of the practising clinicians in our Delphi study, most likely considering the whole severity spectrum. Possibly, this could also at least in part explain why THRT is commonly started at only mild alterations of or even normal TSH (16, 28, 29).

Guidelines are often perceived as universally valid since evidence-based. But, as discussed, the validity of a guideline depends on the validity of the available evidence. The validity of the available evidence, however, depends on the validity of the concept to be examined. The inability of our Delphi panel to achieve consensus on most items and the disagreement with a TSH ≥ 20 mIU/L threshold for treatment suggest that the concept of SCH may not be valid in its current form. It has proven difficult to attribute symptoms associated with SCH to SCH as an underlying cause (30). Thyroid symptoms are mostly non-specific. Therefore, they may lack discriminant potential (31). A Danish study explored thyroid symptoms in 376 individuals with SCH and 7619 euthyroid controls collated from three cross-sectional surveys conducted between 1997 and 2005. This study showed that individuals with SCH did not experience symptoms associated with hypothyroidism more often than euthyroid controls (32).

It has been suggested that TSH concentrations may be more sensitive than thyroid hormone concentrations to a primary change in thyroid function. Defining SCH on the basis of abnormal TSH concentrations would imply that there was a fixed individual pituitary setpoint for TSH, deviation from which would indicate thyroid dysfunction (33). But a fixed TSH set-point could lead to paradoxical situations. For instance, with age, an individual with longstanding stable high normal thyroid hormone concentration could experience decreased thyroid hormone concentrations. If these remained in the normal range, this individual would become more euthyroid, despite a potentially substantial rise in TSH concentration (34). Indeed, fT4 concentrations may align better with clinical parameters (34). A meta-analysis of 58 studies explored the associations between clinical parameters and TSH, fT4 and fT3. Clinical parameters included atrial fibrillation, other cardiac parameters, osteoporosis and fracture, dementia, frailty, mortality, features of metabolic syndrome, and pregnancy outcomes. In this meta-analysis, the clinical parameters were significantly more often associated with thyroid hormone concentrations than with TSH (35). The theory of thyroid hormone concentrations being superior to TSH has, however, also been challenged. A recent study from Denmark followed 20 individuals with SCH and 15 euthyroid individuals with monthly thyroid function tests over one year. This study found TSH to have much higher discriminant value than T4. For T4, the overlap between the SCH and euthyroid group was 92.6%; for TSH, the overlap was 9.0% (36). Another study from the UK, however, examining thyroid tests in 161401 individuals, found that TSH concentrations were not a good discriminant of symptoms attributed to thyroid dysfunction. Furthermore, TSH values depended on age, sex, season and timing of sampling, which would require adjustment of TSH reference ranges (2). Finally, an international online survey of 3915 individuals with self-reported treated hypothyroidism found a 59% prevalence of probable somatic symptom disorder with a tendency to attribute persistent symptoms to hypothyroidism or its treatment (37).

Ultimately, the applicability of a guideline does not only depend on the evidence but also on the context. This has been encapsulated in the postulate, “Globalise the evidence, localise the decision” (38), which can be used as a starting point for translating evidence into clinical practice recommendations (13). The GRADE determinants provide a framework to rate guidelines in terms of evidence and applicability. Bekkering et al., 2019 state that their guidelines adhered to the GRADE format. They state further that the recommendation should not be routinely offered to adults with SCH was strong according to GRADE (8). Most clinicians might agree with that rating. In fact, this concords with our PS 9 regarding the uncertainty about how and when to treat SCH. PS 9 only narrowly missed the 75% threshold for positive consensus. But it would be misleading to extrapolate from the strength of one statement the strength of a whole guideline. Using the GRADE determinants, we could show that recommendations in this area can only be weak at present (panel 1).

Current difficulties to conceptualise SCH as a clinical and pathophysiological entity add to the weakness of currently available guidelines. But current disparities do not invalidate individual illness experience. Unfortunately, disparities regarding diagnosing and treating SCH are likely to persist for the foreseeable future (39). Therefore, clinicians may need to continue to consider each case empirically on an individual basis until our understanding of an underlying thyroid dysfunction improves.

To our knowledge, this is the first study examining attitudes towards diagnosis and treatment of SCH with a Delphi approach. Fifty-three panellists completed all three rounds with a drop-out rate of only 11.7%. A further strength lay in the composition of the panel of the three “stakeholder” specialties, GPs, endocrinologists, and psychiatrists, actively engaged in clinical practice, with equal proportions represented in each round. This allowed an exploration of a difficult area of clinical practice from different perspectives. Finally, the panel was collated from two countries with similar healthcare systems. Even here the panel retained an even split throughout all three rounds. Each subgroup of specialty and country panels comfortably exceeded the minimum recommended number of 10 participants (12).

One major limitation lies in the recruitment of the panellists as a convenience sample. This could lead to an inadvertent selection bias towards like-minded panellists. However, the fact that consensus was only achieved for a few statements, makes such a selection bias unlikely. Use of convenience samples is common in consensus studies and other expert panels formulating clinical guidelines. Relying on random sampling instead might reduce selection bias at the onset of study but increase it during the conduct phase if specialists with stronger opinions were more likely to respond. Also, panellists were recruited according to pre-set criteria. Recruitment from two different countries and three specialists further reduced the scope for selection bias that could ensue from convenience sampling. The relatively large sample size – for a Delphi study – further increased representativeness of the panel.

This study did not involve patients, whose views are equally important. Future work in this area should also include patients. Finally, we did not include questions about the validity of SCH as a clinical or pathophysiological concept. Again, this could be taken up in future work.

## Conclusions

In many aspects, attitudes toward diagnosing and treating SCH remain diverse. Panellists could achieve positive consensus on some diagnostic procedures including the need for repeated testing to establish an SCH diagnosis and the usefulness of antibody screening. However, panellists could not achieve positive consensus on treatment. Regarding negative consensus, a threshold of an TSH of ≥ 20 mIU/L for THRT start, suggested in a previously published guideline, was deemed too high. The inability to achieve consensus on most items during the Delphi process reflects the fact that the scientific evidence in this area is currently not conclusive. Therefore, the guidelines for diagnosing and treating SCH remain weak at present and should not be taken as definite. This may suggest that the concept of SCH needs to be fundamentally rethought with a better understanding of the hypothalamic-pituitary-thyroid physiology. In more general terms, a simple statement that a guideline has been created in GRADE format and providing a one-word summary score regarding its strength may not suffice. Instead, to enable clinicians to estimate the achievable strength of guidelines, a detailed GRADE analysis, addressing all four GRADE determinants, should be provided along newly-created guidelines.

## Data availability statement

The raw data supporting the conclusions of this article will be made available by the authors, without undue reservation.

## Author contributions

IL, CF-C and UW conceptualized the study. All authors participated in the development of the method, recruitment of panelists, formulation of the initial practice statements, and the Delphi investigation. IL and UW analyzed the data and wrote the first draft of the manuscript with support from all other authors. All authors contributed to the article and approved the submitted version.
