# Supplementary material for: Treating subclinical hypothyroidism in individuals with or without mental health problems –A Delphi based expert consensus study in two countries
Source: Front Endocrinol (Lausanne). 2023 Jul 12;14:1204842. doi: 10.3389/fendo.2023.1204842 (PMC10369346; doi:10.3389/fendo.2023.1204842)

**Appendix**

**Questionnaire**

**Treating subclinical hypothyroidism in individuals with or**

**without affective disorder or anxiety –**

**
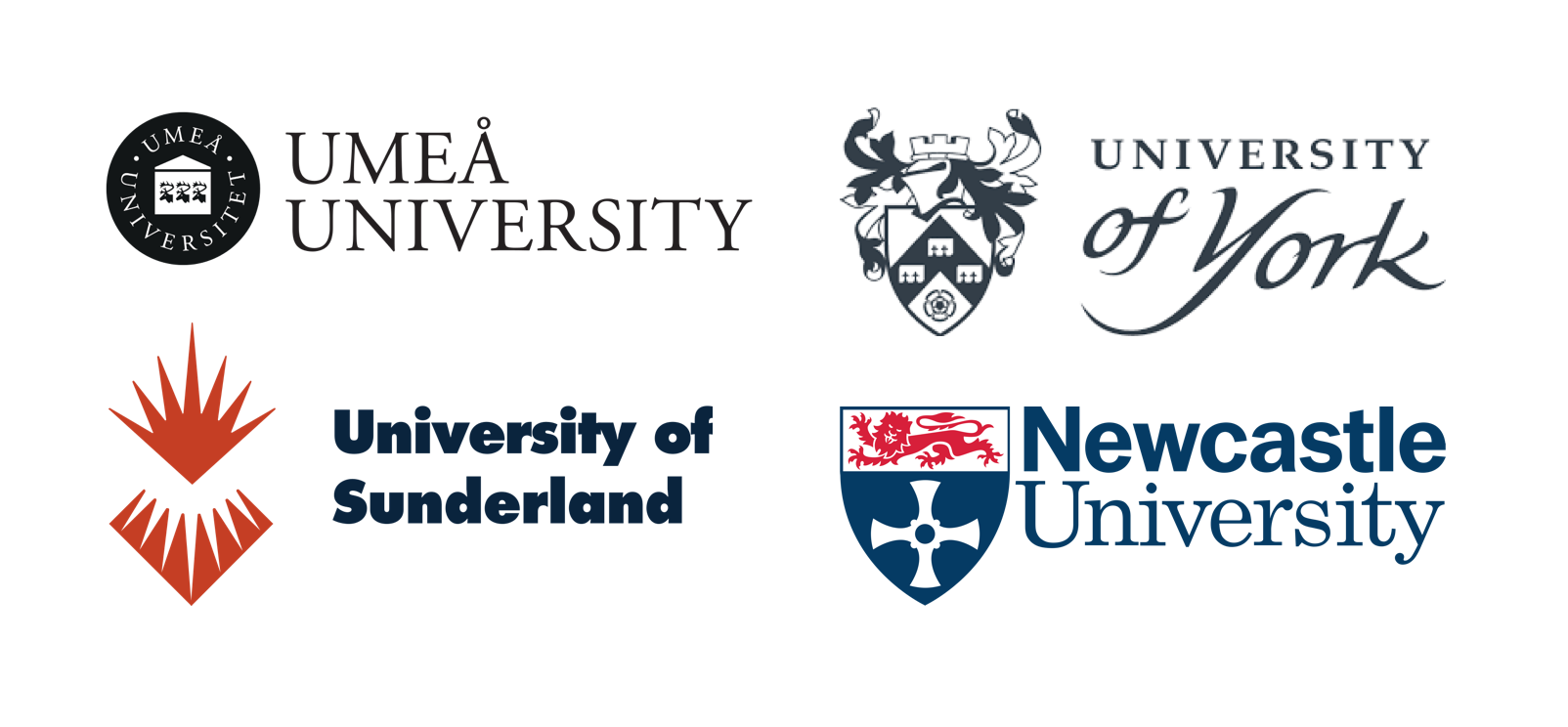
A Delphi based expert consensus study in two countries**

**
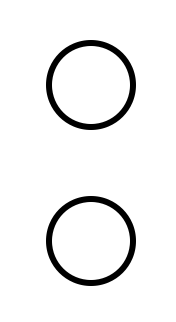
Country**

United Kingdom or experience/knowledge of practice in the United Kingdom

Sweden

**
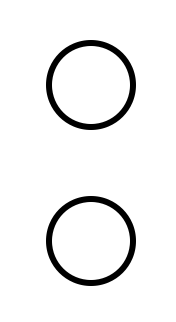
Specialty**

General Practitioner

Endocrinologist

**
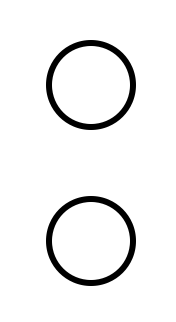
**Psychiatrist

Before you start…
 Please note that

1. This questionnaire is not about right or wrong but about finding out about your attitudes

towards diagnosis and treatment of subclinical hypothyroidism.

2. The practice statements concern subclinical hypothyroidism, established defined as

elevated TSH but normal T4 levels on two occasions.

3. The practice statements concern adults only. The practice statements do not concern

children, adolescents or pregnant women or women trying to become pregnant.

4. We ask separately about individuals with affective disorders and anxiety because they

may be perceived to have different needs.

**Practice Statement 1: In subclinical hypothyroidism, there is often a discrepancy between the laboratory results and clinical symptoms**


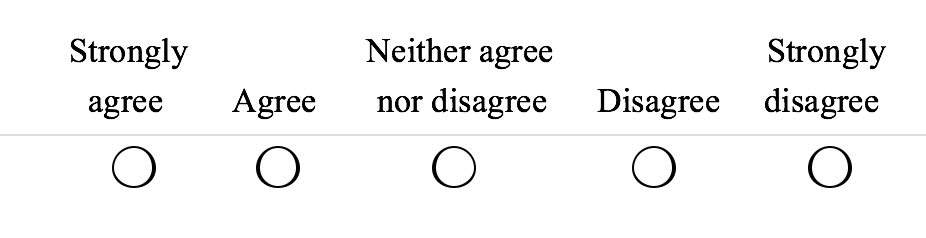


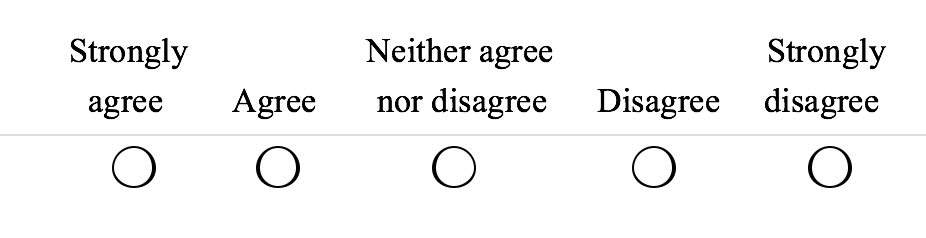
**Practice Statement 2: Depression and fatigue are important symptoms of subclinical hypothyroidism**


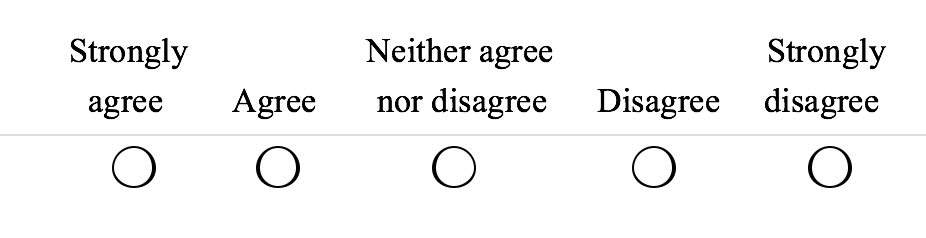
**Practice Statement 3: If TSH is above the reference range but < 10 mIU/L and fT4 is normal, thyroid function tests should be repeated within 3 to 6 months to establish the diagnosis of subclinical hypothyroidism**

**Practice Statement 4: One test with TSH ≥ 10 mIU/L and fT4 normal is not enough to make the diagnosis of subclinical hypothyroidism**


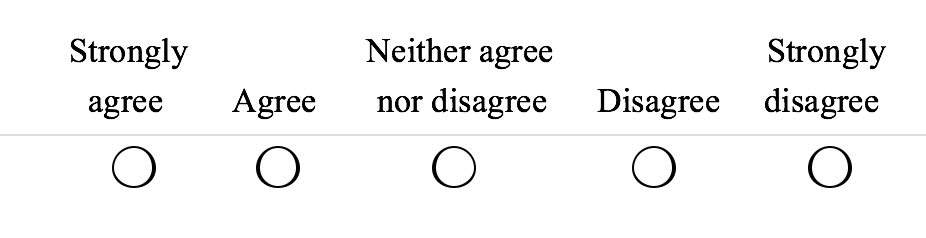


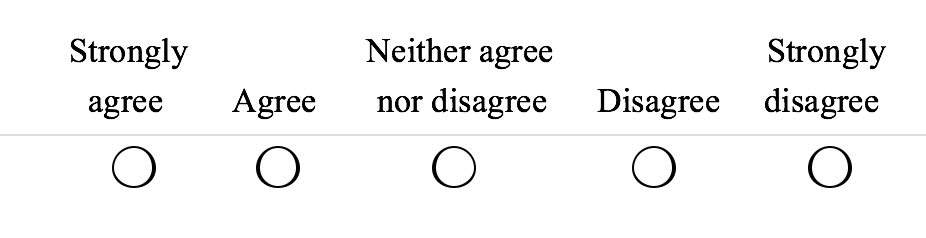
**Practice Statement 5: In subclinical hypothyroidism, psychiatric symptoms are often not recognised**

**Practice Statement 6: Testing for thyroid dysfunction is currently overused in patients presenting with symptoms of depression or anxiety as a main presenting complaint**


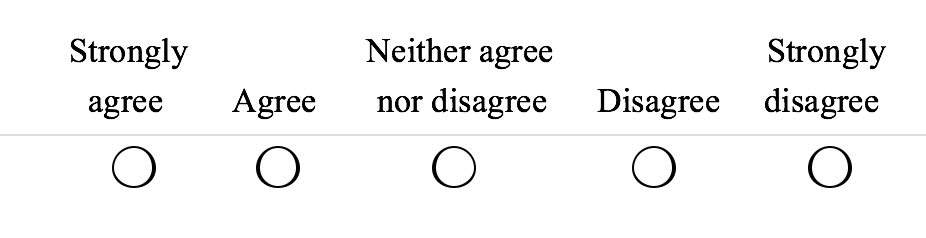


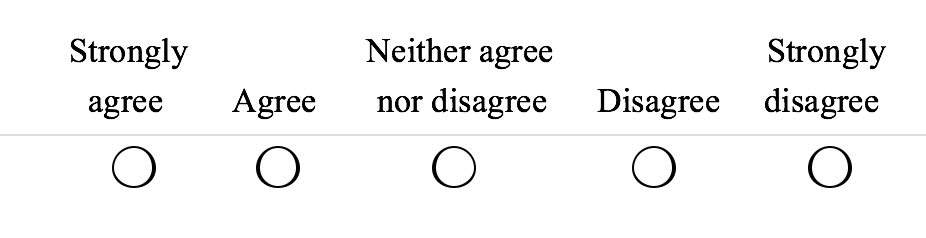
**Practice Statement 7: Testing for thyroid dysfunction is currently overused in patients presenting with fatigue as a main presenting complaint**

**Practice Statement 8: Patients with subclinical hypothyroidism should usually have a thyroid peroxidase (TPO) antibody screen**


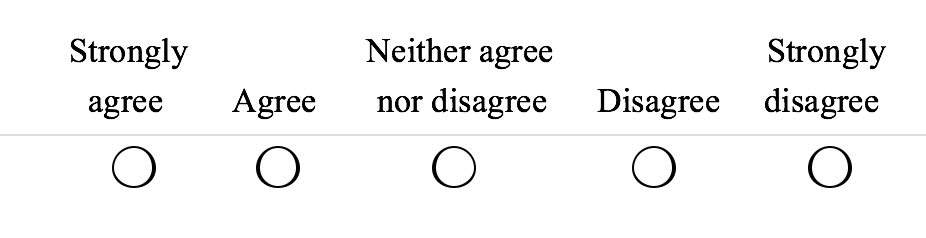


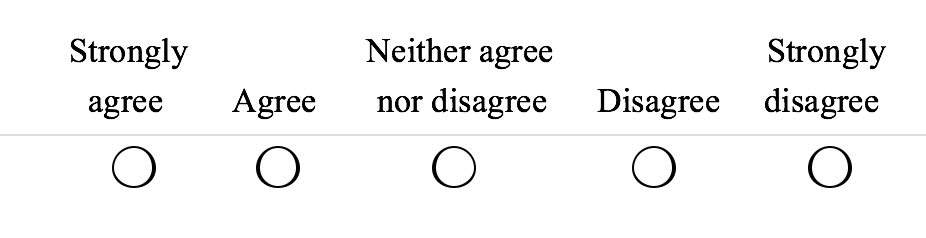
**Practice Statement 9: How and when to treat subclinical hypothyroidism remains unclear**


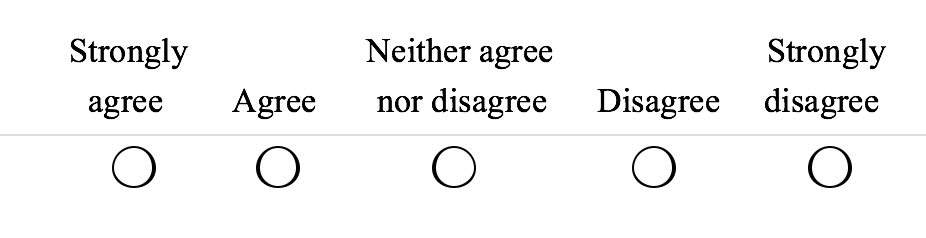
**Practice Statement 10: Laboratory findings weigh heavier than symptoms in the decision to prescribe thyroid hormone replacement therapy forsubclinical hypothyroidism**

**Practice Statement 11: Patients with subclinical hypothyroidism should be offered a trial of thyroid hormone replacement therapy if they report symptoms irrespective of level of TSH, in**

a) individuals without affective disorder
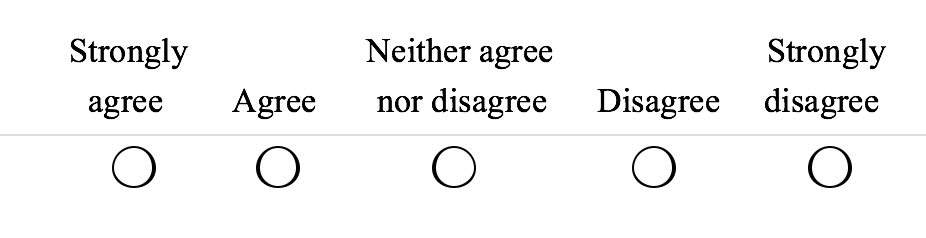
 or anxiety


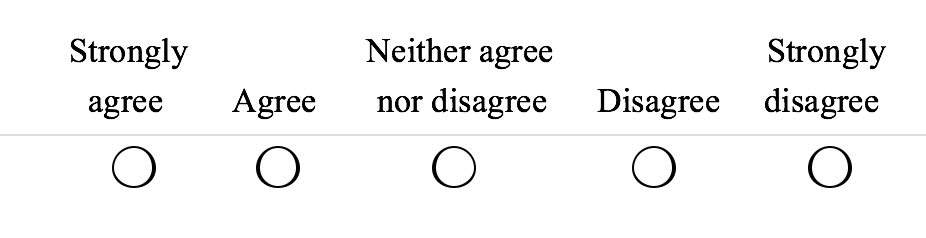
b) individuals with affective disorder or anxiety

**Practice Statement 12: The lowest treatment threshold for prescribing thyroid hormone replacement therapy to patients with subclinical hypothyroidism should be TSH ≥ 10 mIU/L after repeated testing in the absence of TPO antibodies**


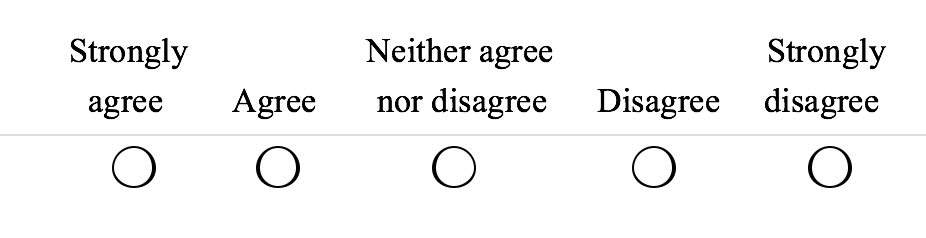


**Practice Statement 13: The lowest treatment threshold for prescribing thyroid hormone replacement therapy to patients with subclinical hypothyroidism should be TSH ≥ 20 mIU/L after repeated testing**


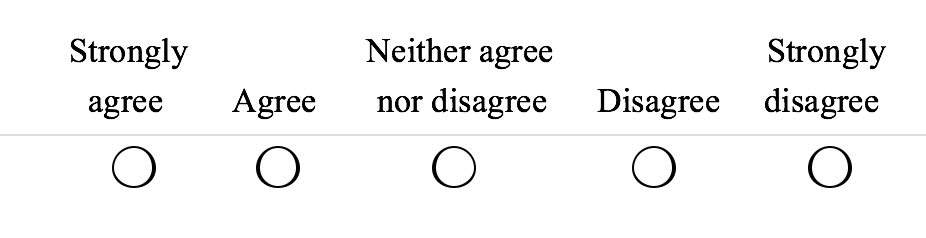


**Practice Statement 14: In older patients (> 70 years of age), there is a risk for overtreating subclinical hypothyroidism**


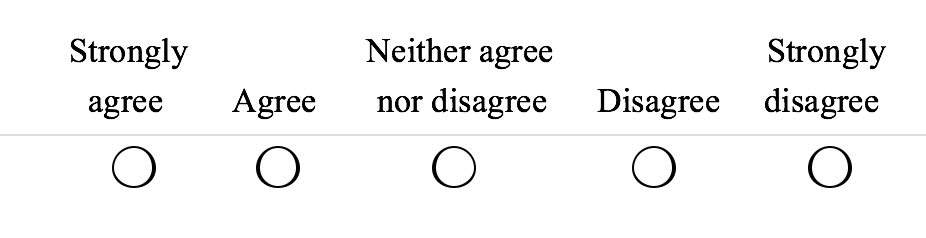


**Practice Statement 15: In patients with subclinical hypothyroidism, presence of TPO antibodies strengthens the indication for thyroid hormone replacement therapy, in**


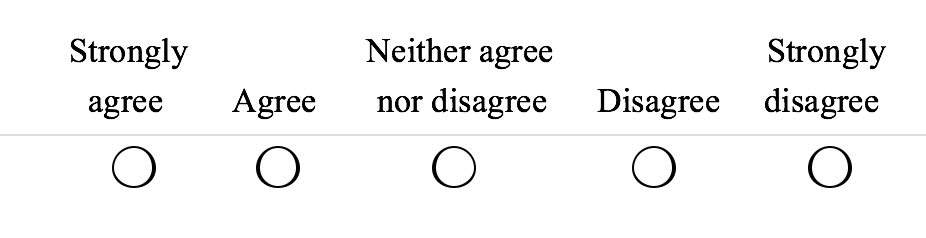
a) individuals without affective disorder or anxiety


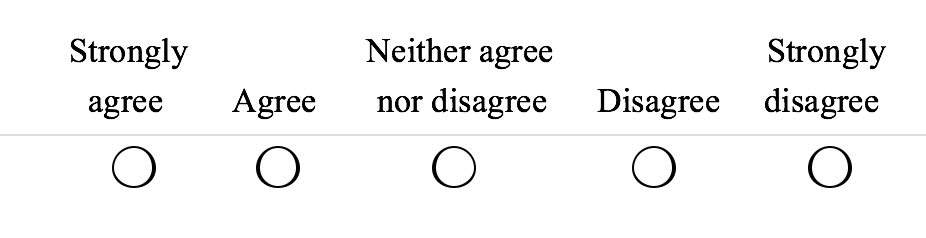
b) individuals with affective disorder or anxiety


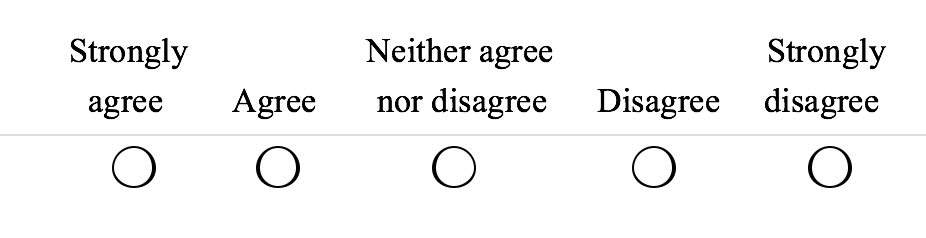
**Practice Statement 16: Subclinical hypothyroidism is currently overtreated**

**Practice Statement 17: If a patient diagnosed with subclinical hypothyroidism asks for a treatment trial, I would arrange it at any raised TSH level on repeated test, even if TPO antibodies are not present, for**

a) individuals without affective disorder or anxiety


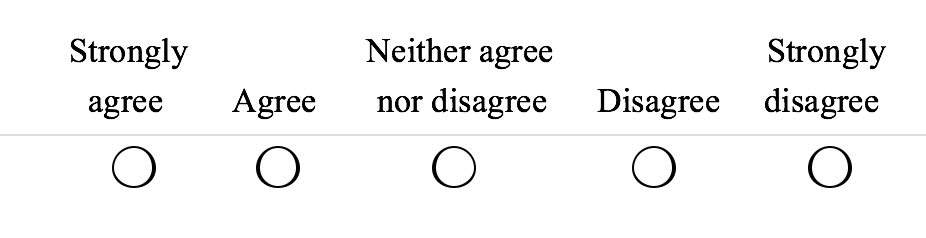


b) individuals with affective disorder or anxiety


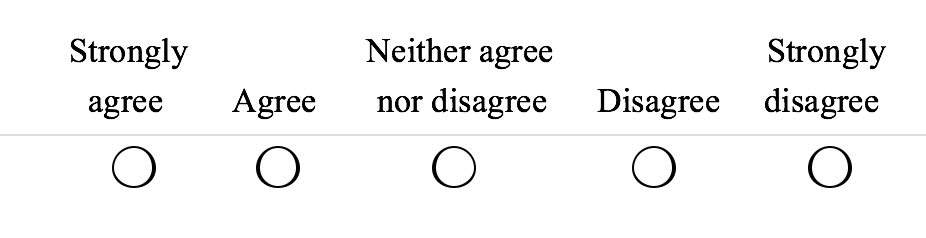


**Practice Statement 18: For deciding on treatment of subclinical hypothyroidism, it is difficult to decide which symptoms are more important: symptoms relating to mental health or symptoms relating to physical health**


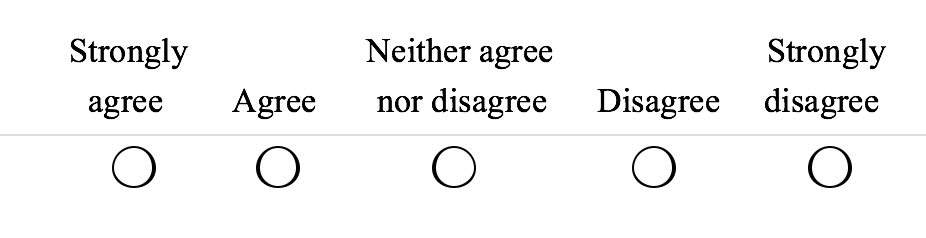


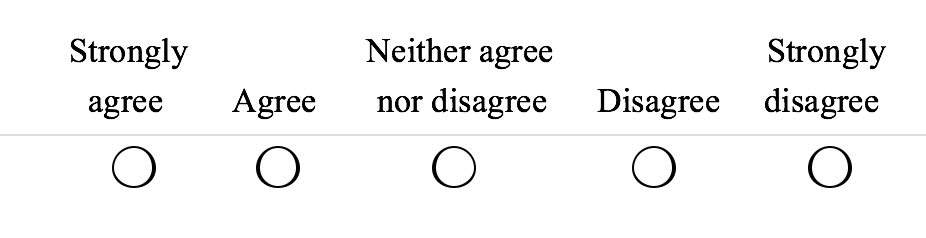
**Practice Statement 19: Cardiovascular and bone health risks influence my decision to treat subclinical hypothyroidism**

**Practice Statement 20: The decision of whether to treat subclinical hypothyroidism can be an important source of conflict between patients and doctors**


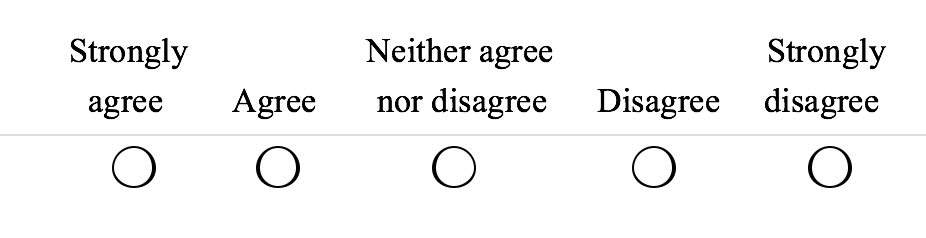


**Practice Statement 21: Patients with subclinical hypothyroidism would benefit from a specialist endocrinology assessment**


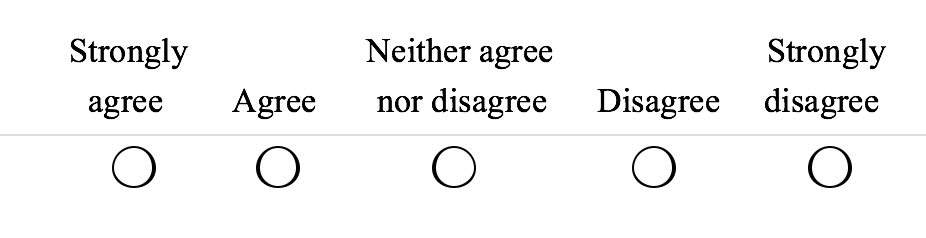


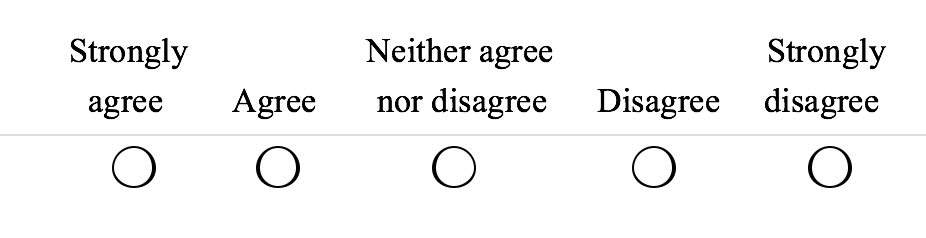
**Practice Statement 22: In patients with subclinical hypothyroidism treated with lithium, the threshold for starting thyroid hormone replacement therapy should be lower**

**Practice Statement 23: In patients with subclinical hypothyroidism treated with antidepressants, the threshold for starting thyroid hormone replacement therapy should be lower**


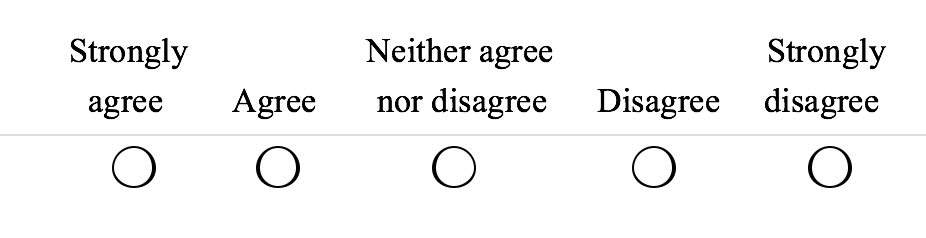

Supplement: Supplementary file 1 [file DataSheet_1.docx]
